# Automated Method for Discrimination of Arrhythmias Using Time, Frequency, and Nonlinear Features of Electrocardiogram Signals

**DOI:** 10.3390/s18072090

**Published:** 2018-06-29

**Authors:** Shirin Hajeb-Mohammadalipour, Mohsen Ahmadi, Reza Shahghadami, Ki H. Chon

**Affiliations:** 1 Department of Biomedical Engineering, Faculty of Medicine, Shahid Beheshti University of Medical Sciences, Tehran 1985717443, Iran; Shirin.hajeb@hotmail.com (S.H.-M.); m_parsi@hotmail.com (M.A.); reza@sbmu.ac.ir (R.S.); 2 Department of Biomedical Engineering, University of Connecticut, Storrs, CT 06269, USA

**Keywords:** automated arrhythmia classification, electrocardiography, health monitoring system, premature ventricular contraction, ventricular fibrillation, atrial fibrillation

## Abstract

We developed an automated approach to differentiate between different types of arrhythmic episodes in electrocardiogram (ECG) signals, because, in real-life scenarios, a software application does not know in advance the type of arrhythmia a patient experiences. Our approach has four main stages: (1) Classification of ventricular fibrillation (VF) versus non-VF segments—including atrial fibrillation (AF), ventricular tachycardia (VT), normal sinus rhythm (NSR), and sinus arrhythmias, such as bigeminy, trigeminy, quadrigeminy, couplet, triplet—using four image-based phase plot features, one frequency domain feature, and the Shannon entropy index. (2) Classification of AF versus non-AF segments. (3) Premature ventricular contraction (PVC) detection on every non-AF segment, using a time domain feature, a frequency domain feature, and two features that characterize the nonlinearity of the data. (4) Determination of the PVC patterns, if present, to categorize distinct types of sinus arrhythmias and NSR. We used the Massachusetts Institute of Technology-Beth Israel Hospital (MIT-BIH) arrhythmia database, Creighton University’s VT arrhythmia database, the MIT-BIH atrial fibrillation database, and the MIT-BIH malignant ventricular arrhythmia database to test our algorithm. Binary decision tree (BDT) and support vector machine (SVM) classifiers were used in both stage 1 and stage 3. We also compared our proposed algorithm’s performance to other published algorithms. Our VF detection algorithm was accurate, as in balanced datasets (and unbalanced, in parentheses) it provided an accuracy of 95.1% (97.1%), sensitivity of 94.5% (91.1%), and specificity of 94.2% (98.2%). The AF detection was accurate, as the sensitivity and specificity in balanced datasets (and unbalanced, in parentheses) were found to be 97.8% (98.6%) and 97.21% (97.1%), respectively. Our PVC detection algorithm was also robust, as the accuracy, sensitivity, and specificity were found to be 99% (98.1%), 98.0% (96.2%), and 98.4% (99.4%), respectively, for balanced and (unbalanced) datasets.

## 1. Introduction

According to the Centers for Disease Control and Prevention, about 735,000 Americans suffer from myocardial infarction every year [1]. Heart disease is one of the leading causes of death, as it accounts for 25% of mortality in the United States every year [2]. It is possible that some of these mortalities can be prevented if malignant arrhythmias are detected early and accurately. With the advent of wearable devices, which can facilitate personalized monitoring, continuous monitoring of cardiac health can now be realized. To this end, more accurate and automated arrhythmia detection algorithms based on electrocardiogram (ECG) signals need to be developed. Arrhythmias can be divided into two main groups. The first group of arrhythmias is not imminently fatal but may require properly diagnosed therapy to prevent morphing into the second group or a serious complication. Arrhythmias in the second group need immediate attention and therapy. Among the various kinds of arrhythmias, ventricular fibrillation (VF) is life threatening [3], while atrial fibrillation (AF) is not, but AF is the most prevalent arrhythmia and increases the risk of stroke [4,5]. Therefore, real-time monitoring and accurate diagnosis of malignant arrhythmia is crucial so that timely preventive treatments can be performed.

Medical devices, such as vital sign monitors, ECG recorders, and Holter monitors, are now routinely available for collection of ECG data. However, other than implantable cardioverters, most are not equipped with automated arrhythmia classification algorithms [6]. Numerous techniques have been reported for arrhythmia classification [6,7,8]. These studies consist of three major phases: preprocessing, extracting features, and classification of various types of arrhythmias in each ECG data segment. The majority of arrhythmia classification is based on the variability of R-R intervals or heart rate variability (HRV) analysis [9,10,11]. (One exception is asystole, which has no R peaks, since the ECG is nearly a flat line.) More recent studies have focused on classification of various arrhythmia patterns [6,12,13,14,15,16,17,18,19,20,21,22,23,24,25,26,27,28,29]. The extracted features for pattern classification have included: (1) time domain or morphology-based features [15,16,17], such as QRS duration and amplitude, P wave duration and amplitude, T wave amplitude, ST segment duration, or QT segment duration; (2) frequency domain features [6,18]; (3) nonlinearity features [19,20,21,22,23]; and (4) rhythm-based features [9,10,11]. Moreover, combining features from various approaches has been shown to improve classification accuracy. For example, wavelet transform (WT) features have been used for classification of arrhythmias with good outcomes [24,25,26,27]. However, some of the issues with WT are the choice of the proper mother wavelet function, appropriate selection of the order of the filter, and the level of signal decomposition. One noteworthy recent approach is variational mode decomposition (VMD), which is based on the modification of the empirical mode decomposition method [13]. The VMD method was shown to provide good classification results, including differentiation of normal (N), premature ventricular contraction (PVC), left-bundle branch block (LBBB), right-bundle branch block (RBBB), premature beats (PB), and atrial premature contraction (APC) beats. While the VMD method was shown to provide good classification results for the rhythms noted above, its capability to differentiate between various segments, such as normal sinus rhythm (NSR), VF, and ventricular tachycardia (VT), was not provided.

**Figure 1:**
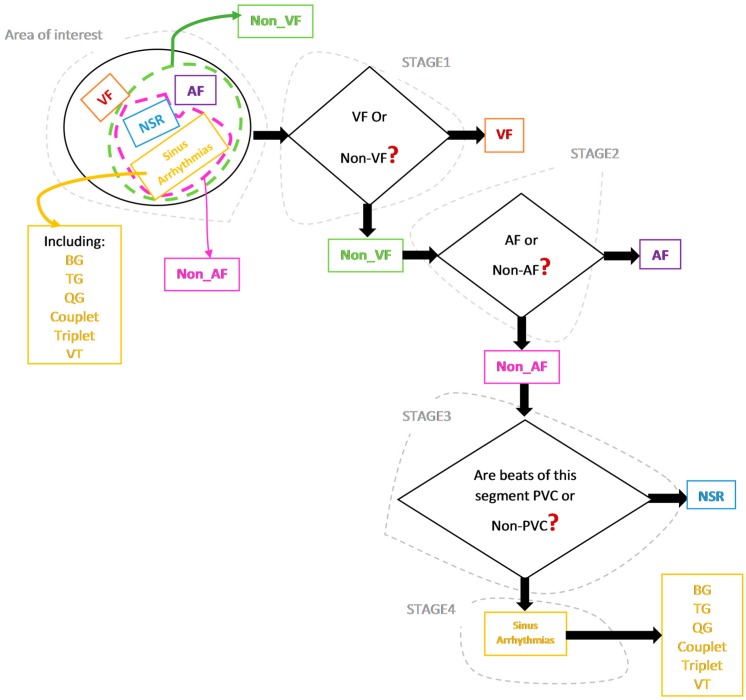
The proposed approach to detect multiple types of malignant arrhythmic episodes. AF: atrial fibrillation; VF: ventricular fibrillation; PVC: premature ventricular contraction; NSR: normal sinus rhythm; BG: bigeminy; TG: trigeminy; QG: quadrigeminy; VT: ventricular tachycardia.

We believe our work is one of the first methods which does not assume the presence of a particular type of arrhythmia. Figure 1 provides our comprehensive approach to detecting various arrhythmias.

We provide a comparison of our algorithm’s performance to the performance of some of the standout algorithms which used the same public databases we did, consisting of the Massachusetts Institute of Technology-Beth Israel Hospital (MIT-BIH) arrhythmia database, Creighton University’s VT arrhythmia database, the MIT-BIH atrial fibrillation database, and the MIT-BIH malignant ventricular arrhythmia database [30].

## 2. Materials and Methods

**Figure 2:**
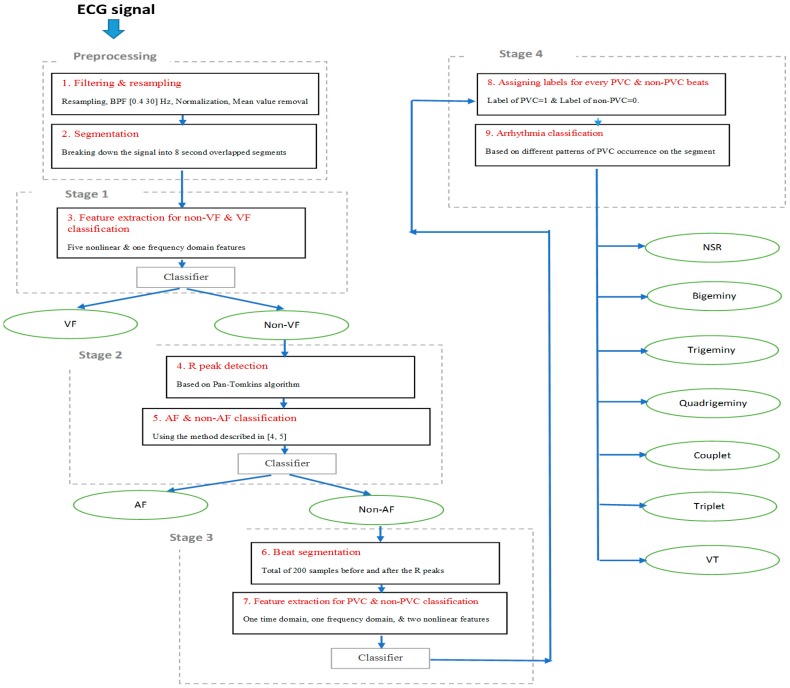
Our overall process.

In real-life scenarios, applications should not be programmed with the type of arrhythmia a patient experiences (they may have no known arrhythmias, and the goal is to detect any that occur). Hence, the purpose of our algorithm development is to screen for various types of arrhythmias, because a wearable device using automated arrhythmia detection will start with no prior knowledge, yet it must be able to find any arrhythmia, or none at all. As shown in Figure 1, the algorithm was designed to be general and identifies various types of arrhythmia a subject may experience. The classification process includes four main stages. Regardless of the arrhythmic type of the input segment, processing starts at stage 1. This stage discriminates between VF and non-VF. The VF arrhythmia can be fatal, thus needs to be detected first. The second stage involves discrimination between AF and non-AF segments using the approach described in [4,5], as well as classification algorithms. The third stage is detection of PVC beats and non-PVC beats (normal (N), atrial premature contraction (APC), premature beats (PB), etc.) in non-AF segments. Finally, the last stage involves finding any repeatable patterns of PVC occurrence in order to perform various sinus arrhythmia classification. Figure 2 is a flowchart of our four-stage classification process, which consists of the following nine steps. We provide the details of each step in this section.

The nine distinct steps:

1. Noise filtering and resampling of signal;
2. Dividing a signal into 8 s segments with 3 s overlapping;
3. Extracting features and training the classifier for distinguishing between VF and non-VF segments;
4. Applying an R peak detection algorithm on each non-VF segment;
5. Extracting features and distinguishing between AF and non-AF segments;
6. Segmenting the beats in every non-AF segment;
7. Extracting features for every single beat and training the classifier for PVC beat detection;
8. Assigning a label for every single beat of every 8 s of signal, based on the results of step 7, in order to detect PVC occurrence patterns;
9. Identifying NSR, bigeminy (BG), trigeminy (TG), quadrigeminy (QG), couplet, triplet, and ventricular tachycardia (VT).

Next, we describe the nine steps, in detail.

### 2.1. Preprocessing/Step 1 and Step 2

All ECG signals are preprocessed as follows:

- Resample each signal with a sampling frequency of 300 Hz;
- Filter the signal with a third-order bandpass Butterworth filter with low cut-off frequency of 0.4 Hz and high cut-off frequency of 30 Hz to suppress baseline drift and reduce the high-frequency noise;
- Convert to physical units, normalize each signal to zero mean and univariance;
- Segment the signal into 8 s data lengths and then shift the data by 3 s, as this provides the best performance [31].

### 2.2. Stage 1/Step 3: Methodology of VF and Non-VF Classification

While there are many algorithms available for automated detection of the onset of VF arrhythmia, there remains room for improvement of VF detection. We describe our approach to discriminate between VF and non-VF segments using six features, of which four are derived from image-based phase plot analysis, one is derived in the frequency domain, and the last reflects the nonlinear characteristics of a data segment. These features are described in detail below.

#### 2.2.1. Image-Based Phase Plot for Morphological Analysis

**Figure 3:**
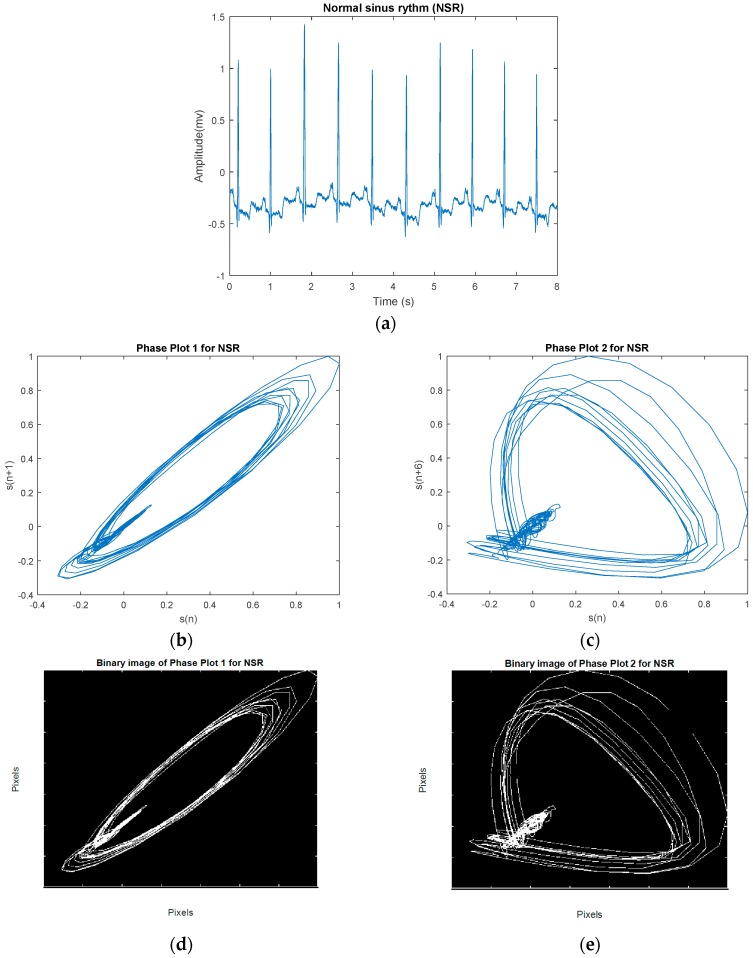
(**a**) An example NSR segment. (**b**) Phase plot of segment with a 1-sample lag. (**c**) Phase plot of segment with a 5-sample lag. (**d**) Related binary image of (b). (**e**) Related binary image of (**c**).

**Figure 4:**
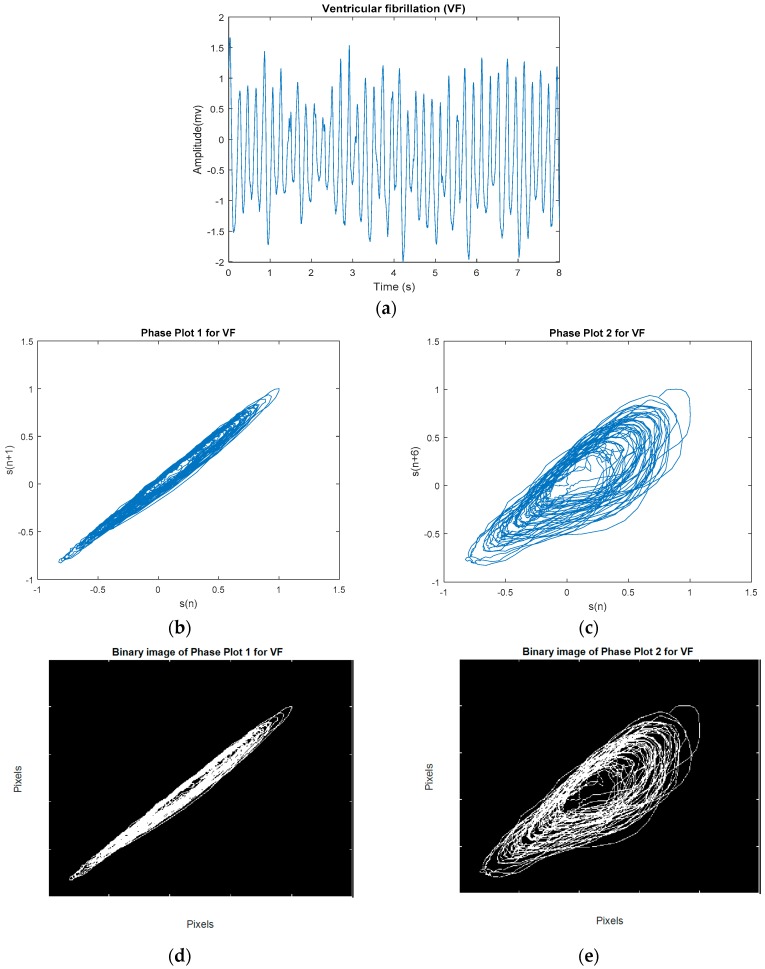
(**a**) An example VF segment. (**b**) Phase plot of segment with a 1-sample lag. (**c**) Phase plot of segment with a 5-sample lag. (**d**) Related binary image of (**b**). (**e**) Related binary image of (**c**).

**Figure 5:**
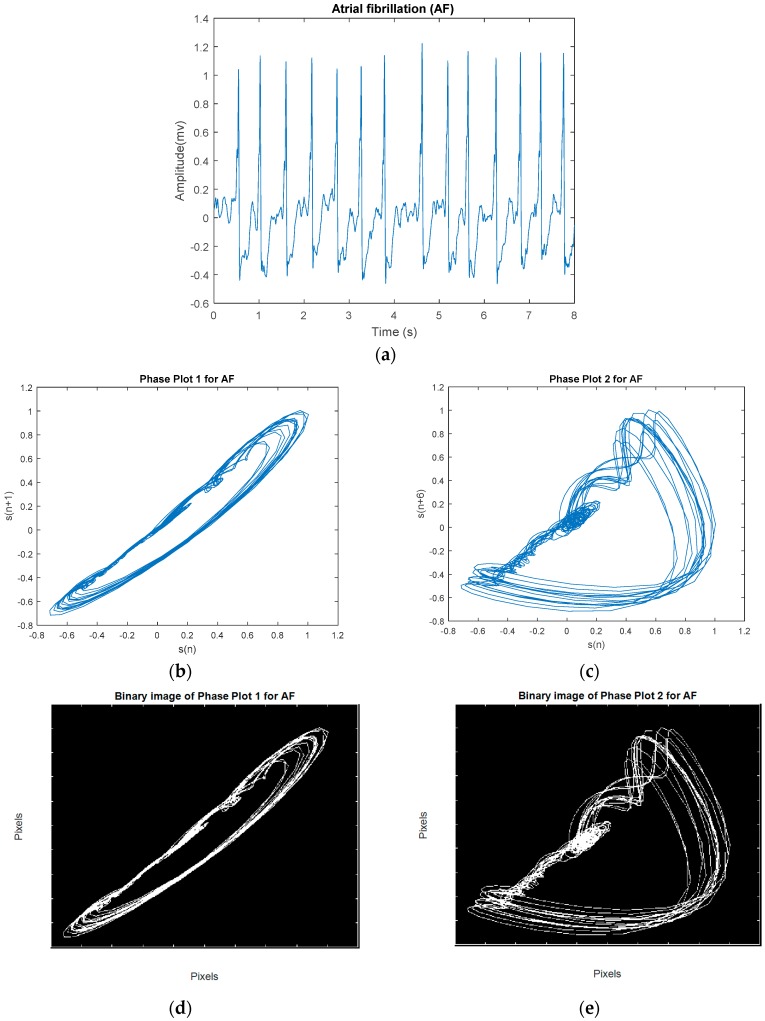
(**a**) An example AF segment. (**b**) Phase plot of segment with a 1-sample lag. (**c**) Phase plot of segment with a 5-sample lag. (**d**) Related binary image of (**b**). (**e**) Related binary image of (**c**).

The phase plot [32] is a simple technique for characterizing nonlinear and non-stationary dynamics of the signal. This is a two-dimensional plot, which unfolds the phase dynamics of the signal if a properly embedded time series in one axis is plotted against the current value of the signal in the other axis. We derived four image-based features according to the distribution and the morphology of the phase plot’s trajectories for classification of VF and non-VF (including AF, NSR, and various other types of sinus arrhythmias). In this study, we constructed two phase plots for each ECG segment. Suppose we have a segment S $\left[s_{1},s_{2},s_{3},\ldots ,s_{n}\right]$, where $s_{i}$ is the *i*th sample. In the first phase plot, the *x* coordinate consists of the current values of $s_{i}$, whereas the y coordinate contains $s_{i+1}$. The second plot visualizes a relation between $s_{i}$ versus $s_{i+5}$; this time delay of 5 samples was chosen to fully unfold the dynamics of the data. Thus, for every data segment, two phase plots with two different time lags are computed. Figure 3 shows a representative NSR segment and its two different time-lagged phase plots. A segment of VF and its two different time-lagged phase plots are shown in Figure 4. While NSR phase plots show organized distinct structures, the VF phase plots show not-well-defined and irregular dynamics, especially with the delay of 5 samples that encompass nearly all of the phase plot. As shown in Figure 3 and Figure 4, the latter figure reflects the less organized behavior of the VF segment in comparison to the NSR segment. Among various types of arrhythmias, VF has the most irregular phase plot, characterized by disorganized trajectories. Phase plots of sinus arrhythmias, such as BG, TG, QG, couplet, triplet, and VT (especially non-sustained VT (NSVT), which is defined as the occurrence of more than 3 consecutive PVCs) are more ordered in comparison to VF. Moreover, AF data have phase plot characteristics similar to an NSR segment, as shown in Figure 5.

**Figure 6:**
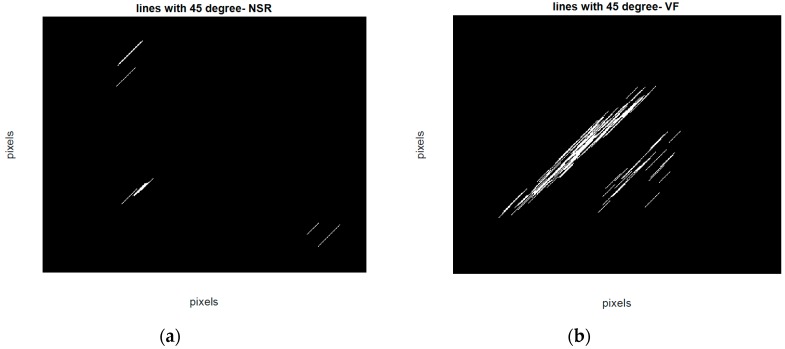
Binary image lines at 45 degrees in the phase plot (**a**) for an NSR segment (**b**) for a VF segment.

To quantitatively discriminate between VF and non-VF from the phase plots, they were subsequently transformed into binary images, which consist of pixels of 1s (white pixels) and 0s (black pixels) [33]. As trajectories of VF phase plots are more randomly distributed in the phase map, it is expected that the number of white pixels in the corresponding binary images will be much greater for VF than for non-VF segments. In order to quantify the differences in the number of white and black pixels, we define S as the total number of pixels in each image and P as the number of corresponding white pixels (number of 1s). A self-similarity index is calculated by taking a ratio of binary pixels in the first phase plot (r1=P1S1) and the same ratio in the second phase plot (r2=P2S2) followed by subtraction of these two ratios ($r_{2}-r_{1}$). The self-similarity index is used as one of the six features and it is denoted as F1 henceforth. It is expected that the self-similarity index value will be smaller for VF than for non-VF, as P (the number of 1s in the binary image) will be greater for the latter, as seen in the binary images in Figure 3e and Figure 4e. The second feature, F2, represents the number of lines in the phase plot’s binary image which are slanted 45 degrees along the diagonal line. Specifically, we assign a value of 1 to a line that is slanted 45 degrees along the diagonal line for a given pixel. To ensure that a line is significantly large, we have a criterion that it must have a length greater than 20 pixels. As shown in Figure 6, some lines are longer than others, but the minimum length is greater than 20 pixels. This threshold was determined heuristically. The VF phase plot would, in general, have a higher number of these 45-degree slanted lines in comparison with the SR segments. F3 reflects the total number of pixels that are occupied by the lines as defined for the feature F2. For example, F3 represents the summation of the lengths of F2 lines. The fourth feature, F4, is extracted through highlighting differences between the number of pixels that span the phase plot of a non-VF segment in comparison with the number of pixels that cover the phase plot of a VF segment. Because of the disorganized behavior of a VF phase plot, the area of white zone in the related binary image is higher than the area of white zone in a non-VF phase plot’s binary image (for instance, compare Figure 3d and Figure 4d). As already mentioned at the beginning of this section, this characteristic was used for extracting F1, which is named self-similarity. In order to better distinguish between non-VF and VF, the new feature (F4) is determined based on these characteristics, using a morphological operator, called morphological filling.

Morphological filling [34], which is an image-processing technique, means encompassing the holes (area of dark pixels surrounded by lighter ones) in a binary or grayscale image. Hence, after subtracting the first and second phase plot images of each segment, a filling operation is applied to the resulting image. Steps for acquisition of this feature are as follows:

- Make the binary image of the first phase plot with delay of 1 for each segment ($B_{1}$)
- Make the binary image of the second phase plot with delay of 5 for each segment ($B_{2}$)
- Subtract $B_{2}$ from $B_{1}$ ($B_{1}$ − $B_{2}$)
- Apply the filling operation on $B_{1}$ − $B_{2}$. (In fact, this operation changes connected background pixels (0s) to foreground pixels (1s), stopping when it reaches object boundaries.)
- Count the number of white pixels or the number of 1s on the resulting binary image.

**Figure 7:**
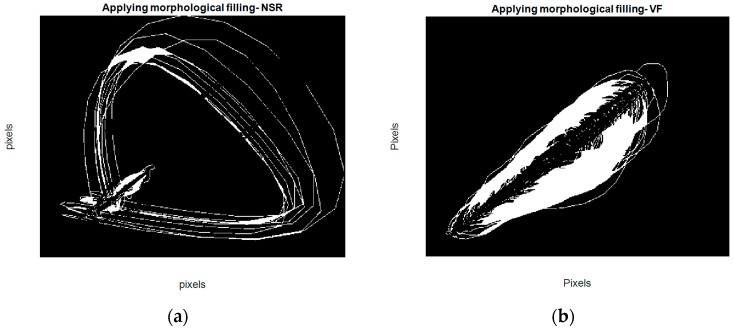
Resulting images for acquisition of the fourth feature (**a**) for an NSR segment (**b**) for a VF segment.

For example, as shown in Figure 7, after the filling operation (which assigns 1 to holes and any other regions which are surrounded by 1) and counting the final number of white pixels (number of 1s), the fourth feature is determined. Note that we used four connected background neighbors for the filling operation. We combined these for image-based features from the phase plot with two more features (a frequency domain feature and Shannon entropy), which are described in the next section, in order to develop a new method for better discrimination between VF and non-VF segments.

#### 2.2.2. Frequency Domain Feature

**Figure 8:**
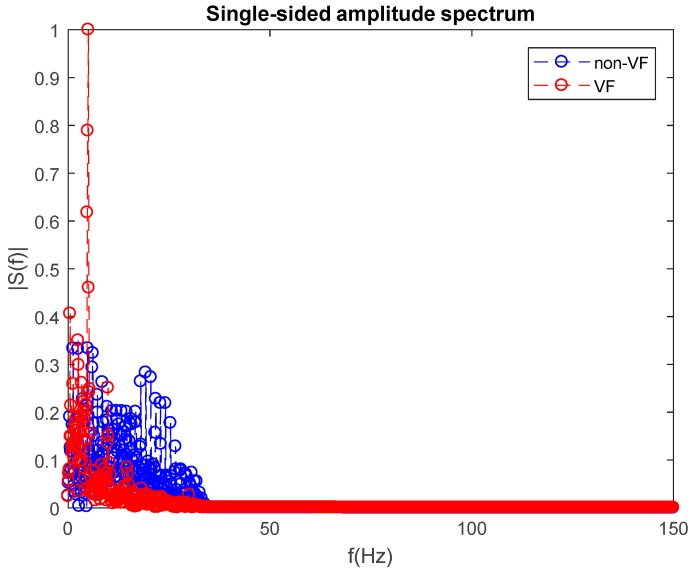
Single-sided amplitude spectra of a non-VF segment (blue) and a VF segment (red).

The fifth feature, F5, for classification of a VF versus non-VF segment, is acquired via analyzing the segment in the frequency domain. Specifically, we used a power spectrum to obtain a classification feature to distinguish between VF and non-VF. Figure 8 shows single-sided amplitude spectra for both VF and non-VF data segments. Each data segment consists of 2400 data points, and we chose a fast Fourier transform data length of 2400 so that the frequency resolution was 0.125 Hz. All amplitudes shown in this figure are normalized to the maximum amplitude. In order to quantify each segment’s spectrum, we calculated the mean spectral amplitude over the entire region. 

As shown in Figure 8, the mean spectral amplitude of a non-VF segment is significantly higher than for a VF segment. This is especially obvious in the frequency range of 0–50 Hz. Counting the number of frequencies which have higher amplitude than the mean value, it is clear that the number of frequencies which have a higher amplitude than the mean value is greater for the non-VF than for VF segments. This can also be seen in Table 1 (feature F5) using the PhysioNet PhysioBank archive datasets. These are described in Section 3.

#### 2.2.3. Nonlinear Feature (Shannon Entropy (SE))

The sixth feature, F6, is based on calculation of the Shannon entropy (SE) of the ECG signal. The SE [35] of signal S is defined as:

(1) $$H\left(S\right)=-\overset{n}{\underset{i=1}{\sum }}p_{i}log_{2}p_{i}$$

The SE value should be higher for VF than for SR, and this was the case for the results shown in Table 1. Finally, using these six features, we determined whether or not an ECG segment is to be classified as either VF or non-VF. The SR segment is then further subjected to PVC detection, when present. 

### 2.3. Stage 2/Steps 4–5: Methodology of AF versus Non-AF Classification

For the steps described in this section, after doing R peak detection [36], we applied the approach described in [4,5] in order to distinguish AF from non-AF segments. The method is based on detecting irregular RR interval characteristics [4,5] and uses a combination of Shannon entropy (SE), turning point ratio (TPR), and root mean square of successive RR differences (RMSSD). In addition, we examined the use of these three features to determine AF detection accuracy using both support vector machines and binary decision tree classifiers. The databases used for distinguishing AF from non-AF segments were the atrial fibrillation database (AFDB) [37] for the former and the MIT-BIH arrhythmia database (MITDB) [38] and the Creighton University ventricular tachyarrhythmia database (CUDB) [39] for the latter.

### 2.4. Stage 3/Steps 6–7: Methodology of PVC and Non-PVC Beat Classification

In the prior two sections, we detailed the procedures for differentiating VF from non-VF episodes followed by differentiating AF from non-AF episodes. In this section, we describe how we detect PVC arrhythmias. The type and pattern of occurrence of PVC beats play an important role in categorizing the type of segment. Therefore, the aim of stage 3 is to identify a PVC beat that might be present, and its pattern of occurrence. For PVC detection, we used ECG signals comprising the lead II configuration from the MITDB.

The PQRS beat segmentation was performed on non-VF and non-AF segments by selecting 200 samples around the QRS complex (70 points to the left side of the R point, 129 points to the right side of the R point). Two hundred samples were selected because we want to cover the entire PQRST complex in all cases including the normal (N) and PVC beats. In normal beats, the intervals of PQ, QRS, and QT are considered as 0.12–0.20, 0.06–0.16, and <0.40 s, respectively. Due to the fact that, in the case of PVC, the duration of QRS can be lengthened by 0.12–0.16 s, the maximum PQRST is around 0.46 s. Thus, with the chosen sampling frequency of 300 Hz for ECG, selecting 200 points or 0.66 s is a reasonable choice.

Four features, including one time-domain feature, one frequency domain feature, and two nonlinear features, were used for classification of N and PVC beats. The four features are described in detail in the following sections.

#### 2.4.1. Time Domain Feature (Local Maxima and Minima Difference (LMMD))

**Figure 9:**
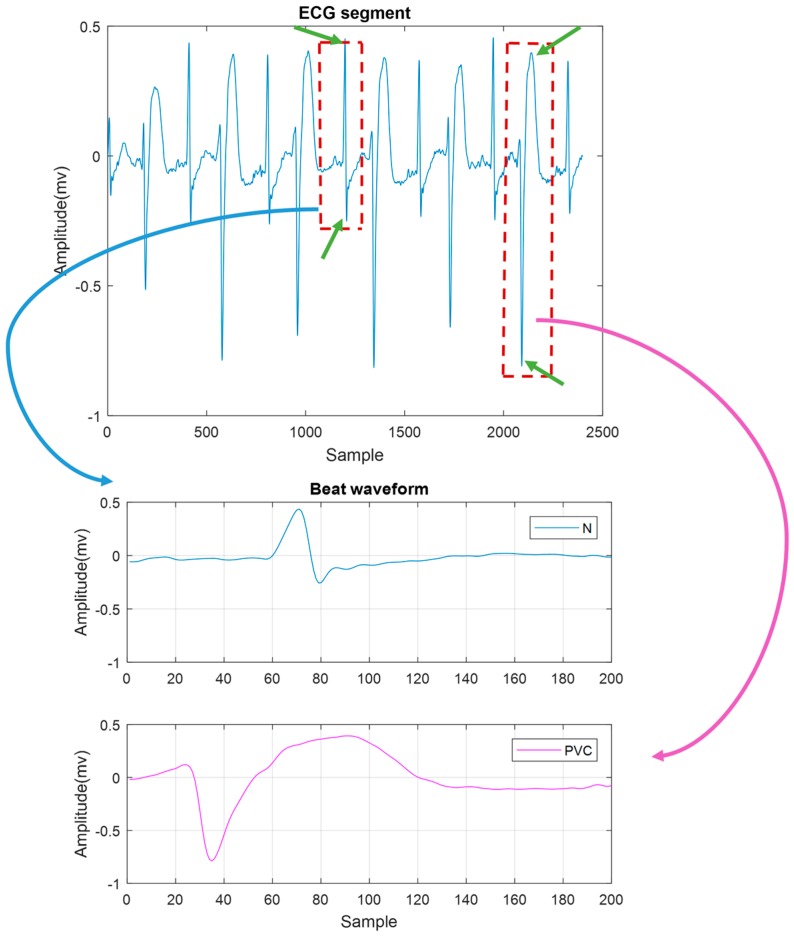
The segment on the top contains a bigeminy episode. On the bottom, the zoomed beats of normal (N) beat (in blue) and PVC (in purple) are shown. The local maxima and minima difference (LMMD) in the PVC beat is much higher than in the N beat. The peaks which we used to calculate LMMD are marked as green arrows.

The time domain feature was extracted based on the distance between a positive and a negative peak on the QRS complex. When PVC occurs, often there is no recognizable P wave prior to the R peak. However, in certain cases it might be observed, and if so, the PR interval is shorter than in NSR. Moreover, a PVC has a long time duration, as shown in Figure 9. For our time domain feature, we calculated the difference between the peak and valley amplitudes in each beat, which we defined as the local maxima and minima difference (LMMD). In a normal beat, the local maximum and minimum are the R and S waves. For a PVC beat, however, we expect the local peak and valley to exhibit a larger LMMD than a normal beat.

#### 2.4.2. Frequency Domain Feature (Maximum Value of Spectral Amplitude (MSA))

**Figure 10:**
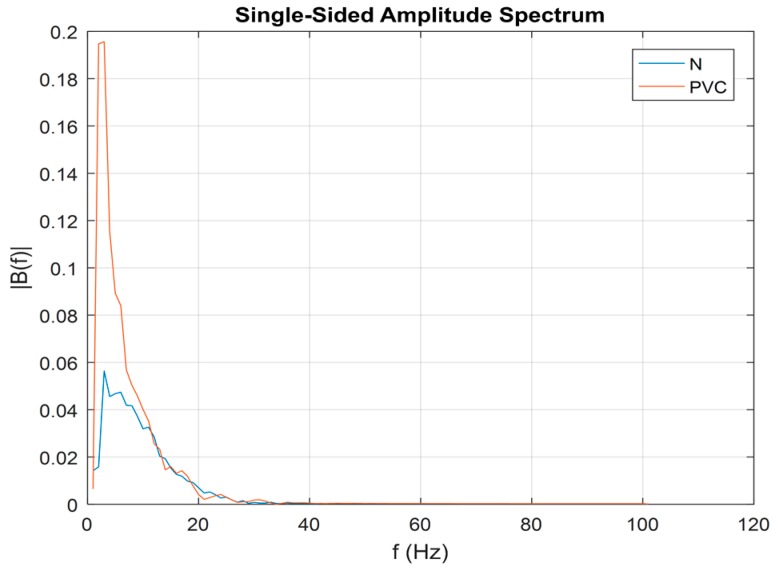
Single-sided amplitude spectrum of normal beat (blue) and PVC beat (red).

We computed the power spectrum of each beat that may contain an N or PVC beat. Representative N and PVC beats’ power spectra are shown in Figure 10. For both rhythms, most of their dynamics are located in the frequency range of 0–20 Hz. However, it is evident that a PVC beat has a larger amplitude value than does an N beat, especially in the low-frequency range (~2–5 Hz). Thus, our second feature for discriminating between PVC and non-PVC beats is the maximum value of the spectral amplitude, and we defined this as the MSA.

#### 2.4.3. Nonlinear Features

We defined two additional features based on nonlinear characteristics of each ECG beat. The first is the wavelet Shannon entropy. The Shannon entropy is used to estimate wavelet coefficients of ECG data.

The second involves calculation of kurtosis (Kurt) of each ECG beat. Kurt is a measure of whether the data are heavy-tailed or light-tailed relative to a normal distribution. The Kurt of a normal distribution is three, and it is defined as:

(2) $$Kurt\left[X\right]=\frac{E\left[{\left(X-\mu \right)}^{4}\right]}{{\left(E\left[{\left(X-\mu \right)}^{2}\right]\right)}^{2}}$$

where μ is the mean value of signal *X*.

### 2.5. Stage 4/Step 8 and Step 9: Methodology of Sinus Arrhythmia Classification

In an arrhythmic segment, PVC beats often occur in repeating patterns. These different patterns produce different types of rhythms, such as: PVC in every other beat (bigeminy: BG), PVC in every third beat (trigeminy: TG), PVC in every fourth beat (quadrigeminy: QG), two consecutive PVCs (couplet), or three consecutive PVCs (triplet). In addition, PVCs that occur for more than three consecutive beats can be classified as ventricular tachycardia (VT). The functional aim of stage 4 is to classify these different patterns of PVC beats. In this section, we describe a simple methodology for sinus arrhythmia episode classification. As detailed in the previous section, beat classification is performed on each segment. Subsequently, by applying a PVC detection algorithm, all beats are classified as either PVC or non-PVC beats. Next, we denote 0 for a non-PVC and 1 for a PVC beat and create a label vector notation, as shown in Table 2. Different patterns of PVCs, as described above, are then noted by the patterns of 1s and 0s in the label vector. Certainly, VT is the most alarming scenario among the various patterns shown in Table 2.

**Figure 11:**
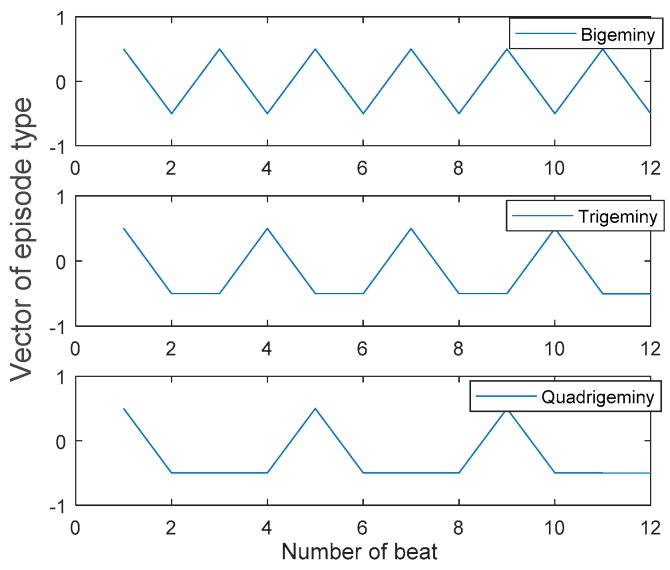
Vector of episode type of three segments with BG, TG, and QG episodes which have 12 beats (Plotted after mean removal).

The following illustrates the sequential steps for classifying NSR, couplet, triplet, and VT:

1. Classify every beat of segment as N or PVC;
2. Label N beats as 0 and PVC beats as 1;
3. Find the number of consecutive PVC beats in each label vector (NCPVC);
4. Classify the label vector as NSR, couplet, triplet, or VT, as shown in Table 3;
5. Differentiate BG, TG, or QG patterns in the data, as shown in Figure 11.

For illustration purposes, suppose that there are 12 beats in a segment. In order to classify data as BG, TG, or QG, the label vector (either 0 or 1) and the locations of the local maxima of the vector are detected.

The label vector patterns for different PVC patterns are illustrated in Figure 11. As shown in this figure, the differences between locations of two consecutive local maxima are 2, 3, and 4 for the cases of BG, TG, and QG, respectively. Thus, based on the differences between the location of the two consecutive local maxima, BG, TG, and QG are classified accordingly. Suppose an ECG signal is comprised of L numbers of 8 s segments with 3 s overlap. The overall proposed method extracts a matrix of 1 by L, which is defined as the vector of episode type (VET). Each element of VET represents a class type of PVC patterns. Table 4 shows the relationship between values of VET and the corresponding episode types.

Therefore, by applying the proposed four-stage approach to an ECG recording, we can derive VET, where every element of this vector represents the arrhythmia type. For example, if the first element VET was 5 and the second was 2, this suggests that the first 8 s of that ECG recording has BG and the second 8 s (with 3 s overlapping) has a couplet.

### 2.6. ECG Databases

The ECG data used in this work came from the MITDB, the CUDB, the MIT-BIH malignant ventricular arrhythmia database (VFDB) [40], and the MIT-BIH atrial fibrillation database (AFDB). We obtained all these data from the PhysioBank archive provided by PhysioNet. The MITDB consists of 48 half-hour recordings of two-channel ECG data obtained from 47 subjects (25 men aged 32–89 years and 22 women aged 23–89 years). The first channel is the lead II recording. All signals were digitized at 360 Hz with 11-bit resolution over a 10 mV range. There is an annotation file for each recording, which contains information on the type of ECG beat and classification of rhythms, including VT, VFL (ventricular flutter), NSR, and so forth. However, due to the lack of VF data in the MITDB, we also used the ECG recordings from the CUDB and the VFDB.

The CUDB and the VFDB consist of 35 8-min and 22 half-hour ECG recordings, respectively. Both databases include data from subjects who experienced episodes of VT, VFL, and VF. All signals were digitized at 250 Hz with 12-bit resolution over a 10 mV range. For the CUDB, the annotation file was marked as “N” for NSR, and other types of arrhythmias, except VF, were also labeled. The approximate start times of VF were also noted in each data segment. Moreover, some portions of data which were unreadable and corrupted were marked as “~” (according to annotation file, some portions are very noisy and unreadable because of very high noise amplitude, very low signal amplitude, loss of signal, or some combination of these).

The VFDB contains two channels of ECG signals for each recording, but we used the lead II signal for data analysis. This database includes 89 episodes of VT, 60 episodes of VFL, and 42 episodes of VF. The annotation labels contain 15 different rhythms, including VT, VFL, VF, NSR, and other rhythms. 

In stage 1 of the evaluation, we used all recordings which had VF portions (according to the annotation files) in the VFDB database (datasets 422, 424, 426, and 430) and CUDB database (datasets CU01, CU04-13, CU15-35). Using these two databases, we analyzed a total of 1691 VF segments (928 from the CUDB and 763 from the VFDB) and 3055 non-VF segments (1255 from the CUDB and 1800 from MITDB databases). These non-VF segments from the two databases (CUDB and VFDB) contain various arrhythmias, including BG, TG, QG, couplet, triplet, VT, and SR. Moreover, to test our discrimination capability between AF and VF in stage 1, we also used the MIT-BIH atrial fibrillation database (AFDB). This database includes 25 10-hour ECG recordings sampled at 250 samples per second. However, we only analyzed 23 out of the 25 recordings for a total of 87,922 AF segments, as datasets 0735 and 3665 do not contain an ECG signal but are only represented by rhythms. The sensitivity, specificity, and accuracy values of the proposed VF detection algorithm are presented in Table 5.

Stage 2 involves AF detection from those data that were identified as non-VF. In other words, this stage involves AF detection versus NSR and various types of sinus arrhythmias. As mentioned earlier, in this stage, the method described in [4,5] was used. In order to make the method applicable to our 8 s segments including various sinus arrhythmias, it was necessary to reset some parameters of the method. For evaluation of this stage, we used 2345 non-AF segments (320 segments from the CUDB and 2025 from the MITDB) and 2345 randomly chosen AF segments from the AFDB.

For the stage 3 evaluation, we used almost all signals with PVC beats in the MITDB dataset for evaluating performance of our PVC classification method. We only discarded those signals with less than 5 PVCs. The MITDB has 7132 PVC beats. We used 7113 PVC beats and 44,794 N beats. Hence, we used recordings 105–109, 114, 116, 118, 119, 124, 200–203, 205, 207, 208, 210, 213–215, 217, 219, 221, 223, 228, 233, and 234.

We partitioned each signal into 8 s segments, because this time length has been reported to be the best choice for classification accuracy [31,41]. Moreover, most automated external defibrillators analyze and display ECG signals in a 6–12 s data segment. While some previously published works [42] have used non-overlapping data segments, we partitioned data into segments overlapping by 3 s, as this allows the most efficient and timely detection of VF when it occurs, in our experience. The motivation for using overlapped segmentation is to recognize a VF portion as soon as it occurs.

### 2.7. Classification

This work contains four classification stages. The first stage is VF versus non-VF segment classification, which is based on features as detailed in Section 2.2. In the second stage, the AF classifier is applied to every non-VF segment to discriminate between AF and non-AF segments [4]. The third classification stage is for separating PVC from non-PVC beats of every non-AF segment, using features described in Section 2.4. The last classification stage discriminates among different types of arrhythmias based on the number and patterns of PVC beats in a given data segment, using the approach detailed in Section 2.5. Training an appropriate classifier is needed for stage 1 and stage 3. For this reason, both training and testing datasets were constructed in two different ways by randomly splitting the dataset. In each case, after extracting features consisting of time and frequency domain features, a vector of features was formulated. This vector was used as an input to the classifier. We performed the classification using both the binary decision tree (BDT) classifier and the support vector machine (SVM). These classification methods need labeled training datasets in order to learn and correctly categorize the untrained data. The SVM is a classifier which first searches for the two closest points of different classes and calls them the support vectors. Then, the decision boundary is determined, which is normal to the support vectors, and the margin between classes is maximized. The slack or *C* parameter, which is needed for the SVM model, was set to 1, and we used a linear kernel. The BDT classifier is a nonlinear mapping method which contains a treelike structure and passes multiple decision steps to obtain a better separation between classes. In this work, we compared the performance of these two classifiers for both classification steps.

For evaluation of classification phases (in stage 1, stage 2, and stage 3), we used two different protocols, producing balanced and unbalanced training and testing processes. Let us consider that we have an A number of samples in class 1 and a B number of samples in class 2.

First protocol: The total number of samples is A + B. We divide this number of samples into two groups of training and testing data. In this protocol, which is called unbalanced dataset, A + B number of samples is randomly split into (A + B)/2 for the training set and (A + B)/2 samples for the testing set. The classification procedure is repeated 5 times. The mean values of the five trials are reported for sensitivity, specificity, and accuracy of the unbalanced dataset.

Second protocol: Let us suppose that A (the number of samples of class 1) is much bigger than B (the number of samples of class 2). In this case, for getting a more balanced classification, we randomly select B samples from class 1 to get equal numbers of samples for both classes. We then randomly split samples into B number of training and B number of testing sets. The classifier is trained on each set. This process is repeated 5 times. We also repeat the entire procedure 5 times, yielding 25 set results in total. The final reported result is the average of each trial.

## 3. Results

For the balanced evaluation of stage 1, an equal number of VF and non-VF segments were used as training and testing datasets. As noted in Section 2.6 (ECG databases), we had 1691 VF segments, 87,922 AF segments, and 3055 arrhythmia segments which are neither VF nor AF. For generating a balanced dataset (described in 2.7), in each trial we randomly chose 1691 cases of segments among the non-VF segments. Both BDT and SVM classifiers were used on the training data to determine classification coefficients. The testing data were then used for evaluation of the performance of both classifiers. For the unbalanced dataset, the entire data of the two groups of VF (1691 episodes) and non-VF were randomly selected and also split equally into training and testing sets.

The averaged results, including standard deviations for differentiation between VF and non-VF segments based on balanced and unbalanced datasets using DT and SVM classifiers, are presented in Table 5. As expected, the balanced datasets provide lower accuracy and specificity but higher sensitivity than the unbalanced datasets for both BDT and SVM classifiers. As shown in Table 5, the accuracy of unbalanced datasets for BDT and SVM are 97.1% and 95.3%, respectively, while these values for balanced datasets are reduced to 95.3% and 90.4%. For both datasets, the BDT classifier provides slightly higher accuracy and sensitivity than does the SVM classifier.

We also evaluated our algorithm for detection of VF against AF segments. The accuracy, sensitivity, and specificity are 96.5%, 96.57%, and 96.45% for balanced BDT and 98.04%, 94.66%, and 98.7% for unbalanced BDT classifiers. Results are shown in Table 6.

**Figure 12:**
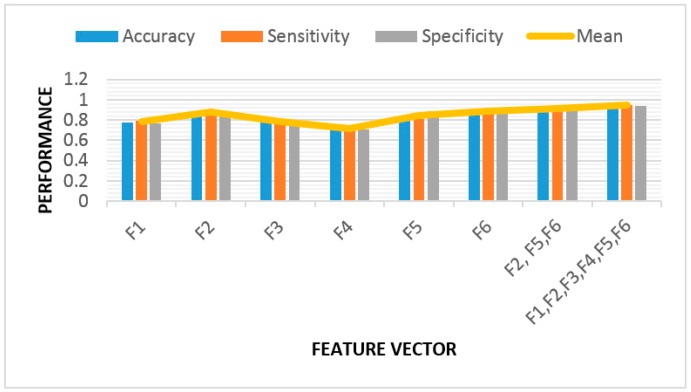
Feature validation for VF detection algorithm based on the balanced binary decision tree (BDT) classifier. The mean is the average of accuracy, sensitivity, and specificity values for each of the feature vectors.

**Figure 13:**
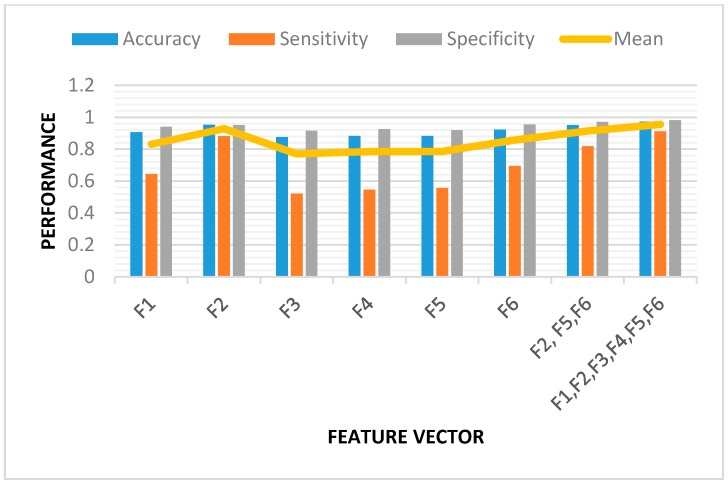
Feature validation for VF detection algorithm based on the unbalanced BDT classifier. The mean is the average of accuracy, sensitivity, and specificity values for each of the feature vectors.

We also evaluated the importance of each feature of our VF detection algorithm, using the BDT classifier on both balanced and unbalanced datasets. The results are shown below in Figure 12 and Figure 13. We analyzed the performance for each feature separately. Based on these results, the most valuable features are F2, F5, and F6. F2 is related to the number of lines at 45 degrees in each subtracted phase plot image, F5 is the spectral feature, and F6 represents SE. Using these three features, the accuracy, sensitivity, and specificity were all found to be 91% for the balanced dataset (Figure 12) using the BDT classifier. The right-most cluster of columns in Figure 12 and Figure 13 show the results after applying all six features.

The AF detection method [4,5] sets threshold values in order to detect VF versus NSR. In stage 2 of our study, we also computed Shannon entropy (SE), turning point ratio (TPR), and root mean square of successive difference (RMSSD) from each 8 s segment. Premature beats have been excluded from AF analysis. Instead of using thresholds for each of the above three features, as was done in [4,5], we trained both BDT and SVM classifiers based on the three features (SE, TPR, and RMSSD). Training a balanced BDT classifier, using 2345 AF segments from the AFDB database and 2345 non-AF segments from both CUDB and MITDB, we obtained 97.8%, 97.21%, and 97.3% as sensitivity, specificity, and accuracy, respectively. The results for the SVM classifier and unbalanced BDT are shown in Table 7. Note the >96% sensitivity, specificity, and accuracy values for both unbalanced and balanced datasets for both classifiers, even when the data length is only 8 s in duration.

The same performance evaluation procedures were applied for PVC detection for the arrhythmic segments (stage 3), using both BDT and SVM classifiers for the balanced and unbalanced datasets. Our database contains 51,907 beats, including 44,794 N and 7113 PVC beats. (The MITDB has 7132 PVC beats. In this work, recordings with fewer than 5 PVC beats were disregarded.) The classification results are represented in Table 8. There was a small difference in the accuracy and sensitivity between the balanced and unbalanced datasets for both BDT and SVM. However, as shown in Table 8, the specificity decreased from 99.4% in the case of unbalanced to 98.4% for balanced datasets for the BDT classifier. In addition, using the SVM classifier, the specificity value decreased from 99.6% in the case of unbalanced to 98.4% for the balanced data. Overall, for both balanced and unbalanced datasets using BDT and SVM classifiers, we obtained high discrimination capability between N and PVC rhythms.

The next classification results involve discrimination among various episodes, including normal SR, BG, TG, QG, couplet, triplet, and VT, for a given signal.

**Figure 14:**
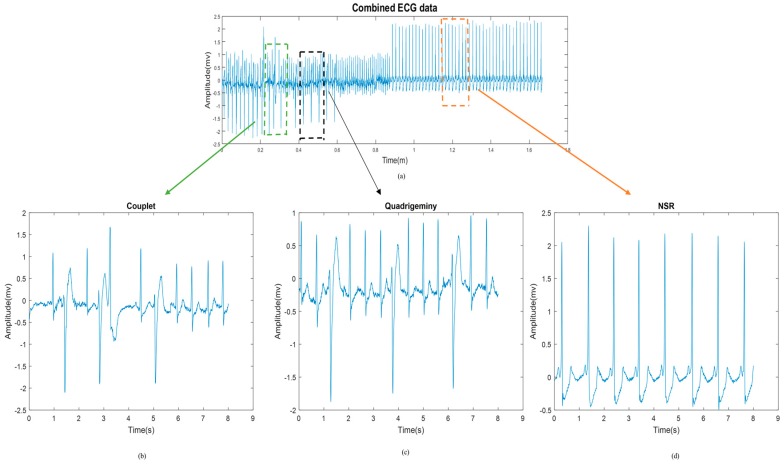
(**a**) Electrocardiogram (ECG) signal including different types of episodes. (**b**–**d**) Zoomed-in episodes of (**b**) couplet, (**c**) QG, and (**d**) NSR.

For each signal, we inserted all of the above-mentioned arrhythmias in random sequences so that we could test the efficacy of our algorithm in detecting these various arrhythmias. Figure 14 shows a representative segment, which exhibits a couplet, QG, and NSR.

In general, the fourth stage’s performance is highly dependent on the performance of our PVC detection method. In fact, as long as our algorithm can recognize PVC beats, it is possible to separate normal SR and different types of arrhythmias based on the label vector of every segment, via the approaches provided in Table 3 and Figure 11 in Section 2.5.

For the last stage of evaluation, 18 randomly selected arrhythmic patterns with a total length of 128 s, contained within 16 segments of 8 s, were sequenced (Figure 14). In total, these signals contain 100 NSR, 40 BG, 40 TG, 20 QG, 40 couplet, 18 triplet, and 30 VT episodes. Table 9 presents the sensitivity in detecting these different types of arrhythmic episodes.

## 4. Discussion

This paper presents a new approach for automated ECG beat and episode classifications, which can be used in wearable devices with no prior knowledge of the wearer’s cardiac health. Note that there is no single database which includes all the types of arrhythmias we focused on. Hence, we used four different databases: the MITDB, CUDB, VFDB and AFDB. All four databases are available from the PhysioNet PhysioBank archive, hence, all investigators have access to them. We believe comparing our method to diverse databases provides a thorough evaluation of the generality of our algorithm.

Our method includes a variety of combined features to classify VF versus non-VF arrythmias. Moreover, our algorithm is also designed to classify various arrhythmias, including AF, BG, TG, QG, couplet, triplet, and VT. In fact, our method is based on segment-by-segment classification followed by beat-by-beat classification. The advantage of our algorithm is that not only can it differentiate between NSR and the two important arrhythmias AF and VF, it can also classify sinus arrhythmias with different patterns of PVC occurrence, which are also known to be clinically important [43,44].

Our work is a comprehensive approach which includes 4 classification stages. We did not use direct classification for two main reasons. First, other published papers have used direct classification, which we have compared our proposed method against, and we have found that our approach provides better results, as stated in this manuscript. Second, and the most important reason for not using direct classification, is that in real-life scenarios, we do not know the type of arrhythmia a patient experiences. Hence, the purpose of our algorithm development was to screen for various types of arrhythmias. The direct classification method is most effective and applicable only when one looks for a specific arrhythmia. For example, if the purpose of the algorithm is to detect sudden cardiac death, then a VT and VF algorithm is all that is needed. However, the purpose of our approach was to discriminate many types of arrhythmias.

If we examine the complexity of each stage separately, the most time-consuming part of the method is stage 1. This is because this stage involves two phase plots with two different time delays for each data segment, followed by image detection. The average running time on an 8 s segment is ~1.07 s for stage 1 (VF classifier), 0.05 s for stage 3 (PVC classifier), and 0.004 s for the last stage (SR classifier) when they are programmed in Matlab 2017a. Thus, the entire execution time for every 8 s of signal is less than 1.15 s using a Dell Precision T3600 computer with an Intel Xeon E5-2667 microprocessor at 2.9 GHz. Certainly, this can be real-time realizable, especially when Matlab code is converted to C or C++ language. Table 10 represents the sensitivity and specificity values of the proposed algorithm for stage 1 versus some other popular approaches. Comparison of this algorithm to five noteworthy algorithms—threshold crossing interval [45], autocorrelation function [46], VF filter [47], spectral analysis methods [48], and the complexity measurement method [49]—is provided in this table.

Table 10 shows that our algorithm provides better performance than some of the popular algorithms. The performance values presented in the table are based on both balanced and unbalanced datasets. It should be noted that for all these methods, the segments with low amplitude, paced beats, significant artifacts, and transitional data from normal to abnormal rhythm were not used. Among the five methods compared, only the VF filter’s performance [47] along with our algorithm yielded sensitivity and specificity values greater than 90%. The other algorithms have either good sensitivity but poor specificity or vice versa. Our approach is the only algorithm that provides both high sensitivity and specificity values, and it has the overall best results when compared to the five other algorithms.

More recently, there have been studies other than those reported in Table 10, which focused on discriminating between shockable (VF/SVT) and non-shockable (non-VF/SVT) rhythms [50,51]. These algorithms’ performance comparison is provided in Table 11. Note that methods that distinguish between VF/VT versus non-shockable arrhythmias (non-VF/VT) tend to have higher performance than methods for classifying VF versus non-VF (e.g., including VT and other non-VF arrhythmias). This is because shockable arrhythmias have different morphological dynamics than do non-shockable arrhythmias, which tend to have more organized patterns. Note that these results are based on an unbalanced data classifier.

In [3], the method is based on a periodogram approach for VF detection. The episode sensitivity of their method was found to be 100%, 90%, and 83% for the MITDB, AHADB, and CUDB datasets, respectively. It should be noted that the MITDB was not used, since it does not have any VF episodes. Table 11 shows other studies for VF detection with different scenarios. All of the reported results have sensitivity and specificity values that are higher than 90%.

As explained in Section 2.2.1, our main features of stage 1 are based on phase plot analysis, which has a higher performance when compared to some of the published methods using a similar phase plot approach [8,33]. Please note that other published works have used Poincare plot VF detection. For example, in [33], only the self-similarity feature extracted from a Poincare plot was used. A threshold value was applied to separate two different classes based on the extracted feature. However, the classification results are lower than our approach, as they found 74.8% for sensitivity and 99.2% for specificity using the MITDB dataset, and sensitivity of 70.2% and specificity of 89.3% for the CUDB dataset. Similarly, in [8], a time delay method was used based on Poincare plots to discriminate VF from sinus arrhythmias. The phase plot method better reflects the arrhythmic differences. However, quantifying these differences to develop features is complicated. Thus, to improve upon these previous works, we also used binary images from each phase plot to obtain characteristics of their phase space trajectories. Moreover, we combined these with a frequency domain feature and Shannon entropy to obtain even better results, and the sensitivity and specificity were found to be 91.1% and 98.2% for the unbalanced and 94.5% and 94.2% for the balanced dataset, respectively.

Stage 2 was to evaluate AF detection. We found that AF detection was accurate using both SVM and DBT classifiers, as the sensitivity and specificity in balanced datasets (and unbalanced, in parentheses) were found to be 97.8% (98.6%) and 97.21% (97.1%), respectively, using the latter approach. These values are similar to AF detection results using 2 min segments, as reported in [4,5].

Stages 3 and 4 are mainly designed to discriminate among various types of arrhythmias (containing PVCs) and NSR. In Table 12, we provide a comparison of our algorithm’s results to some of the published results on PVC detection. These methods, including ours, all used the same MITDB database; hence, fair comparison to our algorithm is possible.

The averaged accuracy, sensitivity, and specificity were found with our method to be 99%, 98.01%, and 98.4%, respectively, for discriminating between the PVC and N beats with the balanced dataset using the BDT classifier; similar results were also found with SVM. As shown, our algorithm far exceeds the performances of the three other algorithms in terms of the accuracy, sensitivity, and specificity.

This integrated algorithm is fast enough to be utilized for real-time processing. In this study, we did not have the opportunity to get real-time signals to report our results against various levels of patient activity. We made an attempt to prepare real-time signals by sequencing distinct types of segments as an input to our algorithm, but all of these segments were acquired from databases which were recorded during rest and do not contain much motion artifact. Although the proposed algorithm is fast enough to be implemented in real-time clinical devices, in order to apply our integrated method in wearable devices, the method would have to be robust against noise, especially motion artifact. In future work, we are interested in applying this algorithm on datasets which contain motion artifact with different levels of activity, such as walking, jogging, or running.

## 5. Limitations

While we stated the aim of the developed algorithm is to provide a comprehensive arrhythmia detection approach for wearable devices, we assumed that a motion artifact detection approach is used to present only the clean portions of data to the algorithm. This approach is reasonable, as wearable devices allow continuous collection of copious amounts of data, hence, it is a feasible strategy to eliminate motion- and noise-corrupted data segments. This is especially important, since the main aim is to have accurate arrhythmia detection while minimizing false positive detection. While we did not provide an approach for motion artifact detection, there have been some recent developments is this active area of research [56,57]. A comprehensive approach will first require accurate detection of motion artifacts, followed by arrhythmia detections on those clean data segments using the various algorithms detailed in this work. 

## 6. Conclusions

In this study, we present a new automated approach for diagnosing and discriminating among various malignant arrhythmias. To verify the excellent performance of the proposed procedure, three different databases were used: the MIT-BIH arrhythmia database, the MIT-BIH malignant ventricular arrhythmia database, and the Creighton University ventricular tachyarrhythmia database, all from the PhysioNet PhysioBank archive. To attain our objectives, we designed a multi-sequential approach with four stages for discriminating among various malignant arrhythmias. Specifically, the new integrated method involves VF, AF, PVC, and sinus arrhythmia episode detection capabilities.

Our approach uses features derived from time-frequency spectral analysis, as well as nonlinear dynamics to discriminate VF from non-VF arrhythmias. Table 10, Table 11 and Table 12 show that classification results were improved using features derived from both approaches. Furthermore, for those identified as non-VF and non-AF, we then detected PVC beats in every sinus rhythm segment based on time domain, frequency domain, and nonlinear features. Eventually, the PVCs and their patterns of occurrence were extracted in order to classify different types of sinus rhythms. We compared the robustness of our approach and found it to be accurate, as the sensitivity, specificity, and accuracy values were all between 92% and 99% in different stages for the databases examined in this work. Furthermore, our approach for VF detection was found to be comparable to most of the published algorithms, while our AF detection showed accuracy values similar to or higher than other AF detection methods, but with a lower data requirement.

This integrated algorithm is found to be fast enough for real-time processing of cardiac signals acquired from wearable devices. In order to apply our integrated method in wearable devices, we need to overcome motion artifacts. There has been much interest in this topic, and some promising results have been reported to mitigate motion artifacts. To this end, we are working on combining motion artifact detection and removal algorithms with our proposed comprehensive arrhythmia detection algorithms.

## Figures and Tables

**Table 1:** Mean ± standard deviation for each feature of the VF detection classifier.

Features	Units	Mean ± STD of Non-VF	Mean ± STD of VF
F1	Ratio	0.02 ± 0.007	0.04 ± 0.01 *
F2	Numbers	7.31 ± 4.22	29.01 ± 10.05 *
F3	Pixels	350.65 ± 390.05	1401.5 ± 786.4 *
F4	Pixels	0.07 ± 0.02	0.12 ± 0.04 *
F5	Numbers	229.06 ± 32.26	157.5 ± 28.33 *
F6	Bits	0.60 ± 0.11	0.86 ± 0.05 *

* Denotes *p* < 0.05 based on Wilcoxon rank sum test, since data were found to be non-normal using the one-sample Kolmogorov–Smirnov test.

**Table 2:** PVC patterns of various episodes.

Episode Type	PVC Pattern
BG	10101010
TG	10010010
QG	10001000
Couplet	11011011
Triplet	11101110
VT	11111111
N	00000000

**Table 3:** Relationship between number of consecutive PVCs (NCPVC) and the type of episode.

NCPVC	Episode Type
0	NSR
≥4	VT
3	Triplet
2	Couplet

**Table 4:** Defining possible values for vector of episode type (VET).

Number	Class Type
1	NSR
2	Couplet
3	Triplet
4	VT
5	BG
6	TG
7	QG

**Table 5:** Performance of stage 1: VF versus non-VF detection.

Classifier	Accuracy	Sensitivity	Specificity	Accuracy	Sensitivity	Specificity
Unbalanced Datasets				Balanced Datasets		
BDT	97.1 ± 0.2%	91.1 ± 1.1%	98.2 ± 0.1%	95.3 ± 0.2%	94.5 ± 0.7%	94.2 ± 1%
SVM	95.3 ± 0.1%	89.5 ± 0.01%	98.2 ± 0.2%	90.4 ± 0.6%	91.6 ± 0.3%	89.3 ± 0.6%

**Table 6:** Performance of stage 1: VF versus AF detection.

Classifier	Accuracy	Sensitivity	Specificity	Accuracy	Sensitivity	Specificity
Unbalanced Datasets				Balanced Datasets		
BDT	98.04 ± 0.00%	94.66 ± 0.01%	98.7 ± 0.00%	96.5 ± 0.03%	96.57 ± 0.05%	96.45 ± 0.00%
SVM	95.26 ± 0.00%	88.79 ± 0.01%	98.74 ± 0.00%	92.35 ± 0.00%	93.41 ± 0.00%	81.34 ± 0.00%

**Table 7:** Performance of stage 2: AF detection.

Classifier	Accuracy	Sensitivity	Specificity	Accuracy	Sensitivity	Specificity
Unbalanced Datasets				Balanced Datasets		
BDT	97.6 ± 0.00%	98.6 ± 0.00%	97.1 ± 0.00%	97.3 ± 0.00%	97.8 ± 0.00%	97.21 ± 0.00%
SVM	97.9 ± 0.00%	98.68 ± 0.00%	95.6 ± 0.00%	96.3 ± 0.00%	98.1 ± 0.00%	97.4 ± 0.00%

**Table 8:** Performance of stage 3: PVC detection.

Classifier	Accuracy	Sensitivity	Specificity	Accuracy	Sensitivity	Specificity
Unbalanced Datasets				Balanced Datasets		
**BDT**	98.1 ± 0.00%	96.2 ± 0.21%	99.4 ± 0.00%	99.0 ± 0.10%	98.0 ± 0.51%	98.4 ± 0.42%
**SVM**	98.4 ± 0.00%	92.0 ± 0.50%	99.6 ± 0.00%	98.1 ± 0.22%	98.1 ± 0.26%	98.4 ± 0.30%

**Table 9:** Stage 4: Evaluation results by type of rhythm.

Class	Total Number	Correctly Classified	True Positive Rate
NSR	100	98	98%
BG	40	39	97.5%
TG	40	37	92.5%
QG	20	20	100%
Couplet	40	36	90%
Triplet	18	17	94.4%
VT	30	27	90%

**Table 10:** Comparison of five popular VF detection algorithms to the proposed method.

Method	SE	SP
TCI	98%	75%
ACF	78%	32%
VF filter	94%	91%
Spectrum	79%	93%
Complexity measure	66%	75%
Proposed method for balanced BDT classifier	94.5%	94.2%
Proposed method for unbalanced BDT classifier	91.1%	98.2%

**Table 11:** Performance of published methods which focus on VF and VT detection vs. our proposed method.

Paper	Scenario	Dataset	SE	SP
[52]	VF/SVT * vs. non-shock	AHA **/CUDB/VFDB	95.93%	94.36%
[53]	VF/SVT vs. non-shock	VFDB	96.03%	93.13%
[52]	VF/SVT vs. non-shock	CUDB	91.21%	93.92%
[50]	VF vs. non-VF	CUDB/VFDB/MITDB	91.9%	97.1%
[50]	VF/VT vs. non-shock	CUDB/VFDB/MITDB	95%	99%
[51]	VF/VT vs. non-shock	AHA/VFDB/CUDB	94.1%	93.8%
Proposed method	VF vs. non-VF	CUDB/VFDB/MITDB/AFDB	94.5%	94.2%
Proposed method	VF vs. non-shock	CUDB/VFDB/MITDB/AFDB	97.6%	97.5%

* Rapid VT or sustained VT (SVT) is defined as more than 180 beats per minute. CUDB: Creighton University ventricular tachyarrhythmia database; VFDB: MIT-BIH malignant ventricular arrhythmia database; MITDB: MIT-BIH arrhythmia database; AFDB: MIT-BIH atrial fibrillation database. ** American heart association (AHA) ECG database

**Table 12:** Performance of some other published methods for PVC detection.

Method	Dataset	Sensitivity	Specificity	Accuracy
Based on wave morphology and R peak [54]	MITDB	94.11%	97.5%	96.45%
Variation of principal directions [43]	MITDB (series 100)	96.12%	---	98.77%
A low-complexity data-adaptive approach [55]	MITDB (series 200)	93.1%	81.44%	98.2%
Proposed method	MITDB	98%	98.4%	99%

## References

[1] Mozaffarian D., Benjamin E.J., Go A.S., Arnett D.K., Blaha M.J., Cushman M., de Ferranti S., Després J.-P., Fullerton H.J., Howard V.J. (2015). Heart disease and stroke statistics—2015 Update. Circulation.

[2] Centers for Disease Control and Prevention Underlying Cause of Death 1999–2014. https://wonder.cdc.gov/wonder/help/ucd.html.

[3] Sadrawi M., Lin C.-H., Lin Y.-T., Hsieh Y., Kuo C.-C., Chien J.C., Haraikawa K., Abbod M.F., Shieh J.-S. (2017). Arrhythmia evaluation in Wearable ECG Devices. Sensors.

[4] Dash S., Chon K.H., Lu S., Raeder E.A. (2009). Automatic real time detection of atrial fibrillation. Ann. Biomed Eng..

[5] Lee J., Reyes B.A., McManus D.D., Maitas O., Chon K.H. (2013). Atrial fibrillation detection using an iPhone 4S. IEEE Trans. Biomed. Eng..

[6] Lin C.H. (2008). Frequency-domain features for ECG beat discrimination using grey relational analysis-based classifier. Comput. Math. Appl..

[7] Zhang L., Guo T., Xi B., Fan Y., Wang K., Bi J., Wang Y. (2015). Automatic recognition of cardiac arrhythmias based on the geometric patterns of Poincare plots. Physiol. Meas..

[8] Roopaei M., Boostani R., Sarvestani R.R., Taghavi M.A., Azimifar Z. (2010). Chaotic based reconstructed phase space features for detecting ventricular fibrillation. Biomed. Signal Process. Control.

[9] Luz E.J., Schwartz W.R., Chávez G.C., Menotti D. (2016). ECG-based heartbeat classification for arrhythmia detection: A survey. Comput. Methods Programs Biomed..

[10] Bai Y., Siu K.L., Ashraf S., Faes L., Nollo G., Chon K.H. (2008). Nonlinear coupling in absence in acute myocardial patients but not healthy subjects. Am. J. Physiol..

[11] Zhong Y., Jan K.M., Ju K.H., Chon K.H. (2006). Quantifying cardiac sympathetic and parasympathetic nervous activities using principal dynamic modes analysis of heart rate variability. Am. J. Physiol..

[12] Mert A. (2016). ECG feature extraction based on the bandwidth properties of variational mode decomposition. Physiol. Meas..

[13] Mert A. ECG signal analysis based on variational mode decomposition and bandwidth property. Proceedings of the 2016 24th Signal Processing and Communication Application Conference (SIU).

[14] Li H., Yuan D., Wang Y., Cui D., Cao L. (2016). Arrhythmia classification based on multi-domain feature extraction for an ECG recognition system. Sensors.

[15] De Chazal P., O’Dwyer M., Reilly R.B. (2004). Automatic classification of heartbeats using ECG morphology and heartbeat interval features. IEEE Trans. Biomed. Eng..

[16] Osowski S., Linh T.H. (2001). ECG beat recognition using fuzzy hybrid neural network. IEEE Trans. Biomed. Eng..

[17] Korürek M., Doğan B. (2010). ECG beat classification using particle swarm optimization and radial basis function neural network. Expert Syst. Appl..

[18] Minami K., Nakajima H., Toyoshima T. (1999). Real-time discrimination of ventricular tachyarrhythmia with Fourier-transform neural network. IEEE Trans. Biomed. Eng..

[19] Afonso V.X., Tompkins W.J. (1995). Detecting ventricular fibrillation. IEEE Eng. Med. Biol. Mag..

[20] Small M., Yu D., Grubb I., Simonotto J., Fox K.A.A., Harrison R. (2000). Automatic identification and recording of cardiac arrhythmia. Comput. Cardiol..

[21] Zhong Y., Wang H., Ju K.H., Jan K.M., Chon K.H. (2004). Nonlinear analysis of the separate contributions of automatic nervous system to heart rate variability using principal dynamic modes. IEEE Trans. Biomed. Eng..

[22] Armoundas A.A., Ju K.H., Lyengar N., Kanters J.K., Saul P.J., Cohen R.J., Chon K.H. (2002). A stochastic nonlinear autoregressive algorithm reflects nonlinear dynamics of heart-rate fluctuations. Ann. Biomed. Eng..

[23] Chon K.H., Mullen T.J., Cohen R.J. (1996). A dual-input nonlinear system analysis of autonomic modulation of heart rate. IEEE Trans. Biomed. Eng..

[24] Lin C.H., Du Y.C., Chen T. (2008). Adaptive wavelet network for multiple cardiac arrhythmias recognition. Expert Syst. Appl..

[25] Salah H., Noureddine E. (2015). Cardiac arrhythmia classification by wavelet transform. Int. J. Adv. Res. Artif. Intell. (IJARAI).

[26] Alickovic E., Subasi A. (2016). Medical decision support system for diagnosis of heart arrhythmia using DWT and random forests classifier. J. Med. Syst..

[27] Lopez A.D., Joseph L.A. Classification of arrhythmias using statistical features in the wavelet transform domain. Proceedings of the 2013 International Conference on Advanced Computing and Communication Systems (ICACCS).

[28] Ge D., Srinivasan N., Krishnan S.M. (2002). Cardiac arrhythmia classification using autoregressive modeling. Biomed. Eng. OnLine.

[29] Tripathy R.K., Sharma L.N., Dandapat S. (2016). Detection of shockable ventricular arrhythmia using variational mode decomposition. J. Med. Syst..

[30] Goldberger A.L., Amaral L., Glass L., Hausdorff J.M., Ivanov P.C.H., Mark R.G., Mietus J.E., Moody G.B., Peng C.K., Stanley H.E. (2000). PhysioBank, PhysioToolkit, and PhysioNet: Components of a New Research Resource for Complex Physiologic Signals. Circulation.

[31] Amann A., Tratnig R., Unterkofler K. (2005). Reliability of old and new ventricular fibrillation detection algorithms for automated external defibrillators. BioMed. Eng. OnLine.

[32] Brennan M., Palaniswami M., Kamen P. (2001). Do existing measures of Poincare plot geometry reflect nonlinear features of heart rate variability?. IEEE Trans. Biomed. Eng..

[33] Amann A., Tratnig R., Unterkofler K. (2007). Detecting ventricular fibrillation by time-delay methods. IEEE Trans. Biomed. Eng..

[34] Soille P. (1999). Morphological Image Analysis: Principles and Applications.

[35] Shannon C.E. (1948). A mathematical theory of communication. Bell Syst. Tech. J..

[36] Pan J., Tompkins W.J. (1985). A real-time QRS detection algorithm. IEEE Trans. Biomed. Eng..

[37] Moody G.B., Mark R.G. (1983). A new method for detecting atrial fibrillation using R-R intervals. Comput. Cardiol..

[38] Moody G.B., Mark R.G. (2001). The Impact of the MIT-BIH Arrhythmia Database. IEEE Eng. Med. Biol..

[39] Nolle F.M., Badura F.K., Catlett J.M., Bower R.W., Sketch M.H. (1986). CREI-GARD, A new concept in computerized arrhythmia monitoring systems. Comput. Cardiol..

[40] Greenwald S.D. (1986). The Development and Analysis of a Ventricular Fibrillation Detector. Master’s Thesis.

[41] Verma A., Dong X. (2016). Detection of ventricular fibrillation using random forest classifier. J. Biomed. Sci. Eng..

[42] Li Q. (2014). Ventricular fibrillation and tachycardia classification using a machine learning approach. IEEE Trans. Biomed. Eng..

[43] Zarei R., He J., Huang G., Zhang Y. (2016). Effective and efficient detection of premature ventricular contractions based on variation of principal directions. Digit. Signal Process..

[44] Alajlan A.Y., Bazi F., Malek M.S. (2014). Detection of premature ventricular contraction arrhythmias in electrocardiogram signals with kernel methods. Signal Image Video Process.

[45] Thakor N.V., Zhu Y.S., Pan K.Y. (1990). Ventricular tachycardia and fibrillation detection by a sequential hypothesis testing algorithm. IEEE Trans. Biomed. Eng..

[46] Chen S., Thakor N.V., Mower M.M. (1987). Ventricular fibrillation detection by a regression test on the autocorrelation function. Med. Biol. Eng. Comput..

[47] Kuo S., Dillman R. (1978). Computer detection of ventricular fibrillation. IEEE Comput. Cardiol..

[48] Barro S., Ruiz R., Cabello D., Mira J. (1989). Algorithmic sequential decision-making in the frequency domain for life threatening ventricular arrhythmias and imitative artefacts: a diagnostic system. J. Biomed. Eng..

[49] Zhang X.S., Zhu Y.S., Thakor N.V., Wang Z.Z. (1999). Detecting ventricular tachycardia and fibrillation by complexity measure. IEEE Trans. Biomed. Eng..

[50] Atienza F.A., Morgado E., Martínez L.F., Alberola A.G., Álvarez J.L.R. (2014). Detection of life-threatening arrhythmias using feature selection and support vector machines. IEEE Trans. Biomed. Eng..

[51] Jekova I. (2007). Shock advisory tool: Detection of life-threatening cardiac arrhythmias and shock success prediction by means of a common parameter set. Biomed. Signal Process. Control.

[52] Jekova I., Krasteva V. (2004). Real time detection of ventricular fibrillation and tachycardia. Physiol. Meas..

[53] Jekova I. (2000). Comparison of five algorithms for the detection of ventricular fibrillation from the surface ECG. Physiol. Meas..

[54] Detection of Premature Ventricular Contraction Beats Using ANN. http://connection.ebscohost.com/c/articles/82678089/detection-premature-ventricular-contraction-beats-using-ann.

[55] Li P., Liu C., Wang X., Zheng D., Li Y., Liu C. (2014). A low-complexity data-adaptive approach for premature ventricular contraction recognition. Signal Image Video Process..

[56] Lee J., McManus D.D., Merchant S., Chon K.H. (2012). Automatic motion and noise artifacts detection on Holter ECG data using empirical model decomposition and statistical methods. IEEE Trans. Biomed. Eng..

[57] Chong J., Dao D.K., Salehizadeh S.M.A., McManus D.D., Darling C.E., Chon K.H., Mendelson Y. (2014). Photoplethysmograph signal reconstruction based on a novel hybrid motion artifact dection-reduction approach- Part I: Motion and noise artifact detection. Ann. Biomed Eng..

